# Targeting basal-like breast tumors with bromodomain and extraterminal domain (BET) and polo-like kinase inhibitors

**DOI:** 10.18632/oncotarget.14465

**Published:** 2017-01-03

**Authors:** Cristina Nieto-Jiménez, Ana Alcaraz-Sanabria, Javier Pérez-Peña, Verónica Corrales-Sánchez, Gemma Serrano-Heras, Eva M. Galán-Moya, Leticia Serrano-Oviedo, Juan Carlos Montero, Miguel Burgos, Juan Llopis, Atanasio Pandiella, Alberto Ocaña

**Affiliations:** ^1^ Translational Research Unit, Albacete University Hospital, Albacete, Spain; ^2^ Cancer Research Center, CSIC-University of Salamanca, Salamanca, Spain; ^3^ Centro Regional de Investigaciones Biomédicas (CRIB), Universidad de Castilla La Mancha, Albacete, Spain

**Keywords:** breast cancer, triple negative breast cancer, polo-like kinases, BET inhibitors, JQ1

## Abstract

Metastatic triple negative breast cancer (TNBC) is an incurable disease with limited therapeutic options, and no targeted therapies available. Triple negative tumors and the basal-like genomic subtype, are both characterized by a high proliferation rate and an increase in cell division. In this context, protein kinases involved in the mitotic formation have a relevant role in this tumor subtype. Recently, Bromodomain and extraterminal domain (BET) inhibitors have shown to be active in this disease by modulating the expression of several transcription factors. In this article, by using an *“in silico”* approach, we identified genomic functions that can be inhibited pharmacologically in basal-like tumors. Functional annotation analyses identified “cell division” and “regulation of transcription” as upregulated functions. When focus on cell division, we identified the polo-like kinase 1 (PLK) as an upregulated kinase. The PLK inhibitor Volasertib had the strongest anti-proliferative effect compared with other inhibitors against mitotic kinases. Gene expression analyses demonstrated that the BET inhibitor JQ1 reduced the expression of kinases involved in cell division, and synergized with Volasertib in a panel of triple negative cell lines. Combination of both agents augmented cell death. Similarly, combination of both compounds reduced the expression of stem cell markers. Globally, this data demonstrates the synergistic interaction between BET and PLK inhibitors, paving the way for their future clinical development.

## INTRODUCTION

Basal-like breast tumors and its clinical correlate the triple negative breast cancer (TNBC) are both an unmet disease, where no therapeutic strategies exist beyond treatment with classical chemotherapy [1]. Due to its heterogeneity the identification of relevant druggable targets has been unsuccessful and no novel compounds have reached yet the clinical setting [2, 3]. In this context, the discovery of relevant non-oncogenic vulnerabilities that could be therapeutically exploited is a main goal.

Although heterogeneous, this breast cancer subtype is characterized by some common clinical aspects, including sensitivity to chemotherapy, poor prognosis, and a specific relapse pattern [3, 4]. From a biological point of view, triple negative breast tumors have a high proliferation rate, have significant genetic instability and present deficiencies in genes involved in the control of the DNA damage response [5–8]. Due to its high proliferation this tumor subtype is more sensitive to agents that act on functions related to cell division such as chemotherapies or novel anti-mitotic agents [9–12]. In this context, it has been suggested that agents against kinases that participate in the mitotic process could have a relevant place in the future clinical armamentarium [13].

Bromodomain and extraterminal (BET) inhibitors are a new family of compounds that by targeting bromodomains regulate the expression of transcription factors (TFs) [14, 15]. In contrast to diseases where a specific TFs is the key oncogenic event, like in neuroblastoma; in tumors with a high grade of heterogeneity, like TNBC, the modulation of the expression of several TFs can affect different functions, some of which may be associated with an oncogenic phenotype. Our group, among others, has demonstrated that epigenetic modulators, like BET inhibitors have a profound antitumor activity in TNBC *in vitro* and *in vivo* [16, 17]. The way BET inhibitors produce their antiproliferative effect is complex, and involve the inhibition of several TFs that subsequently affect cell division [16, 17].

We hypothesized that agents that act on epigenomic events like BET inhibitors, could indirectly regulate important pathways required to sustain proliferation or survival, by modulating the expression of several genes involved in those processes. In addition, those agents could be used to boost the action of targeted agents that are currently approved or in clinical development.

In this article by using an *in silico* approach we identified several kinases involved in the G2/M cell cycle phase that could be inhibited pharmacologically. The BET inhibitor JQ1 reduced the expression of several of them and synergized with the polo-like kinase inhibitor Volasertib. JQ1 arrested cells at G1 but when combined with a polo-like kinase inhibitor induced a mitotic catastrophe that led to cell death. Of note the association of both agents had an increased effect on the stem cell properties of the tumoral cells.

Taken together our results demonstrate that the combination of epigenetic agents with specific kinase inhibitors targeting dependent non-oncogenic functions like mitosis is a potential therapeutic approach, and support the development of agents that affect non-oncogenic vulnerabilities in tumors with a high grade of heterogeneity.

## RESULTS

### Functional transcriptomic analyses of basal-like tumors identify druggable kinases involved in mitosis

**Figure 1:**
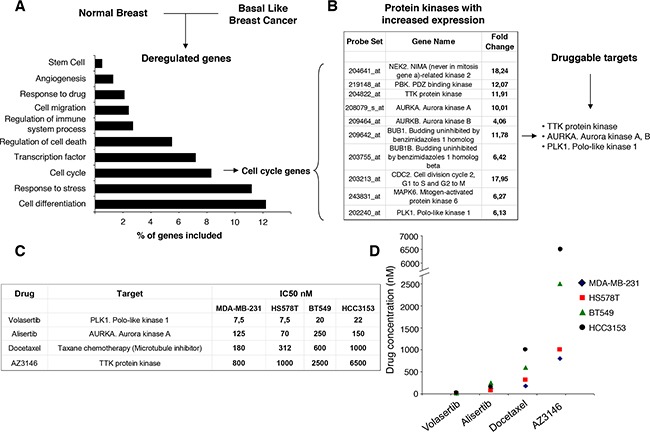
Identification of druggable cell cycle kinases in basal-like breast cancer and antitumoral activity of mitotic kinase inhibitors **A**. Comparison of gene expression profiles in basal-like tumors versus normal breast tissues identified differentially regulated genes involved in several functions. The bars indicate the percentage of total modified genes in each cellular activity. **B**. Table of selected genes coding for cell cycle kinases that display higher expression (with a > 4 fold change) in breast cancer than in normal breast. A list of 3 druggable mitotic kinases is also indicated. **C, D**. Determination of the half maximal inhibitory concentration (IC50, nM) for selected experimental drugs (Volasertib, Alisertib and AZ3146) targeting mitotic kinases.

To identify genes that are differentially expressed in normal epithelial cells compared with basal-like tumors, we performed gene expression analyses using a public dataset [18, 19]. Functional clustering analyses revealed several deregulated functions involved in oncogenic transformation (Figure 1A), including cell cycle, cell differentiation, response to stress or regulation of transcription, and some of them can be inhibited pharmacologically.

Genes included in the cell cycle function were next evaluated. Among them, ten transcripts coding for protein kinases were involved in the regulation of cell division or mitosis (Figure 1B). These genes were not upregulated in the same amount in other breast cancer subtypes (Supplementary Table 1). Of note, polo-like kinase 1, aurora kinases A and B, and mps1/TTK are druggable kinases for which novel kinase inhibitors are currently in clinical development [9–13].

### Pharmacologic screening identifies the polo-like kinase inhibitor Volasertib as an active agent

Based on the druggable kinases identified, a pharmacologic screening with agents in clinical development was performed against these proteins. We used Alisertib as an aurora kinase A and B inhibitor, AZ3146 as a mps1/TTK inhibitor and Volasertib as a polo-like kinase inhibitor. In addition, we used docetaxel, an approved anti-tubulin chemotherapy, as a control. As can be seen in Figure 1C and 1D the polo-like kinase inhibitor Volasertib showed a clear anti-proliferative activity, with a more favorable IC50 compared with the other agents.

### The polo-like kinase inhibitor Volasertib synergizes with BET inhibitors

**Figure 2:**
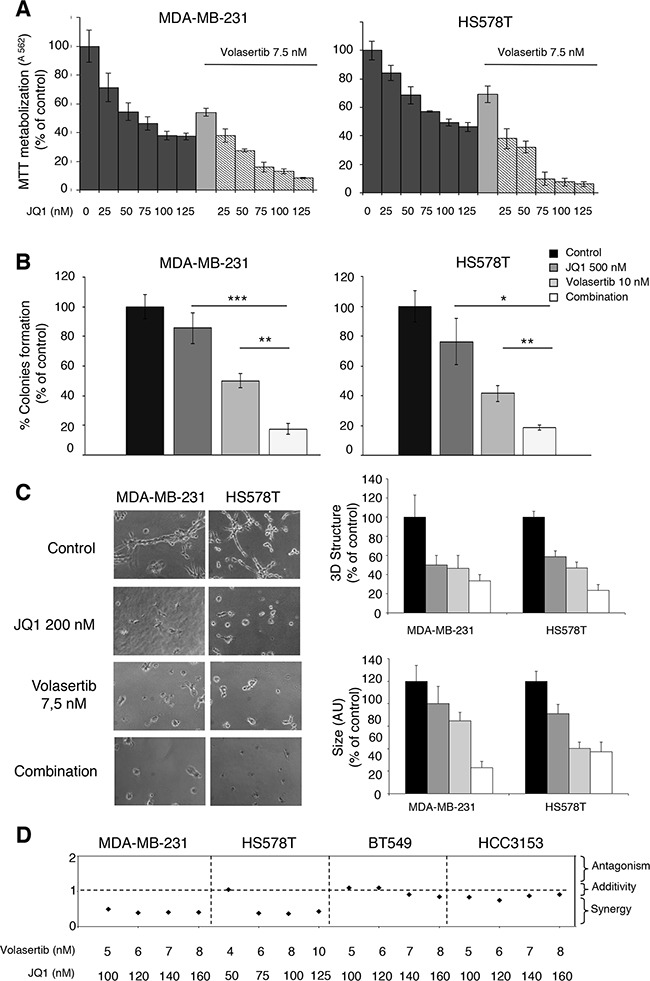
Synergistic action of polo-like kinase inhibitor and BET inhibitor on proliferation of triple-negative breast cancer cells **A**. Effect of the combination of Volasertib and JQ1 in triple-negative breast cancer cells. MDA-MB-231 and HS578T cells were incubated with the indicated concentration range of the compounds, and after 3 days of drug exposure, metabolization of MTT in viable cells was determined by spectrophotometry. The mean absorbance values of untreated cell lines (controls) were taken as 100%. **B**. Action of Volasertib and JQ1 on colony formation. TNB cells were treated with each inhibitor (Volasertib 10 nm, JQ1 500 nM) alone and in combination for 5 hours. Then, cells were washed, subjected to serial dilution 1/10 and seeded. After 10 days of incubation, the number of colonies were determined. **C**. Capacity of cell growth in semi-solid medium in the presence of polo-like kinase and BET inhibitors. MDA-MB-231 and HS578T cells were plated in 48 multiwell plates and grown in medium containing matrigel for 3 days in the presence of JQ1 (200 nM) alone and combined with Volasertib (7,5 nM). All images were taken at ×20 magnification. The quantitation of sphere diameter was performed manually by tracing a straight line across the sphere diameter of untreated cells (controls) and scoring its value as arbitrary length units. Total number of colonies per plate was manually counted. Data of three experiments (MMT assay, clonogenic experiment and Matrigel 3D cultures) are represented as the mean ± s.d. of triplicates. *p<0.5; **p<0.005; ***p<0.001. **D**. Quantitation of synergistic anti-proliferative effect of Volasertib and JQ1 in breast cancer cells. Combination indexes for the different drug combinations were obtained using CalcuSyn program and plotted.

As observed in our genomic analyses (Figure 1A), regulation of transcription was an altered function in this tumor subtype, and agents targeting transcription factors like BET inhibitors have shown activity in TNBC [16, 17]. In this context, we decided to evaluate the efficacy of novel BET inhibitors in combination with the polo-like kinase inhibitor Volasertib. We explored the interaction of both agents in MDA-MB231 and HS578T, observing that increasing doses of the BET inhibitor JQ1 augmented the effect of Volasertib given at its IC50 dose (Figure 2A).

An increase in the activity of the combination compared with each agent alone was further observed in clonogenic assays, or when evaluating the activity using a semi-solid media with matrigel, although to a lower extent compared with MTTs assays, particularly for HS578T (Figure 2B, C).

Finally, we observed a synergistic interaction in MDA-MB231, HS578T, BT549 and HCC3153 with the combination of both agents (Figure 2D).

### JQ1 reduces the expression of mitotic-kinases and induces a mitotic catastrophe in combination with the polo-like inhibitor

**Figure 3:**
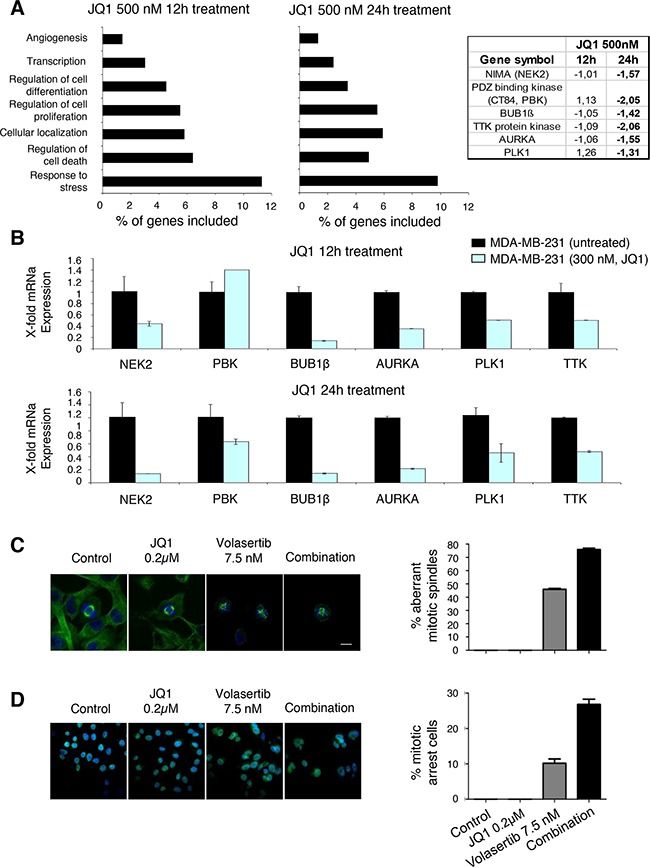
Downregulation of mitotic kinases by BET inhibitor and Induction of mitotic catastrophe with the combination **A**. Identification of genes whose transcription levels is altered in MDA-MB-231cells after incubation with JQ1 (500nM) for 12 and 24 hours using Gene-set enrichment analysis and DAVID Bioinformatics Resources 6.7. Fold change levels from gene expression analyses of protein kinases associated with mitosis regulation, including NIMA, PDZ binding kinase, BUB 1β, TTK, AURKA and PLK1 are shown. **B**. mRNA levels of selected genes were determined by quantitative rt-PCR in TNB cell line before and 12, and 24 hours after treatment with JQ1, at a concentrations of 300nM. PCR conditions and primer sequences are described in “Material and Methods”. **C**. Aberrant mitotic spindles caused by treatment with JQ1 and Volasertib in MDA-MB-231 cells. Fluorescence images showing β-tubulin immunoreactivity (green) and DNA staining (blue) were obtained by confocal microscopy. **D**. Control and JQ1-treated cells showed a typical mitotic spindle in mitotic cells, while aberrant spindle formation could be observed in cells treated with Volasertib, or JQ1 + Volasertib. Scale bar = 10 μm. Quantification of the percentage of aberrant mitotic spindles is shown in the histogram. Number of mitotic cells: n=33 (ctrl), 32 (JQ1), 30 (Volasertib), 26 (JQ1 + Volasertib).

To uncover the potential causes behind the synergistic interaction between JQ1 and Volasertib, we first evaluated the effect of JQ1 using transcriptomic analyses. To do so, we treated MDA-MB231 cells with the BET inhibitor JQ1 and extracted mRNA at 12 and 24 hours. Functional analyses identified cell proliferation as one of the affected functions (Figure 3A). The specific assessment of genes involved in mitosis reveled that NIMA, PDZ, BUB1β, TTK, Aurora A and PLK1 were reduced by JQ1; all these kinases are involved in the regulation of cell cycle. qPCR experiments confirmed the reduction of the identified genes (Figure 3B). These data suggest that treatment with JQ1 acts on several functions, and cell cycle control is one of the affected.

Next, we assessed the effect of both compounds on mitosis by staining β-tubulin and DNA, and evaluating them using confocal microscopy. Control and JQ1-treated cells showed a typical mitotic spindle, while aberrant spindle formation could be observed in cells treated with Volasertib; that was increased with the combination (Figure 3C). The combination also produced a mitotic arrest, compared with each agent given alone (Figure 3D).

### Pleiotropic effect of both agents on cell cycle

**Figure 4:**
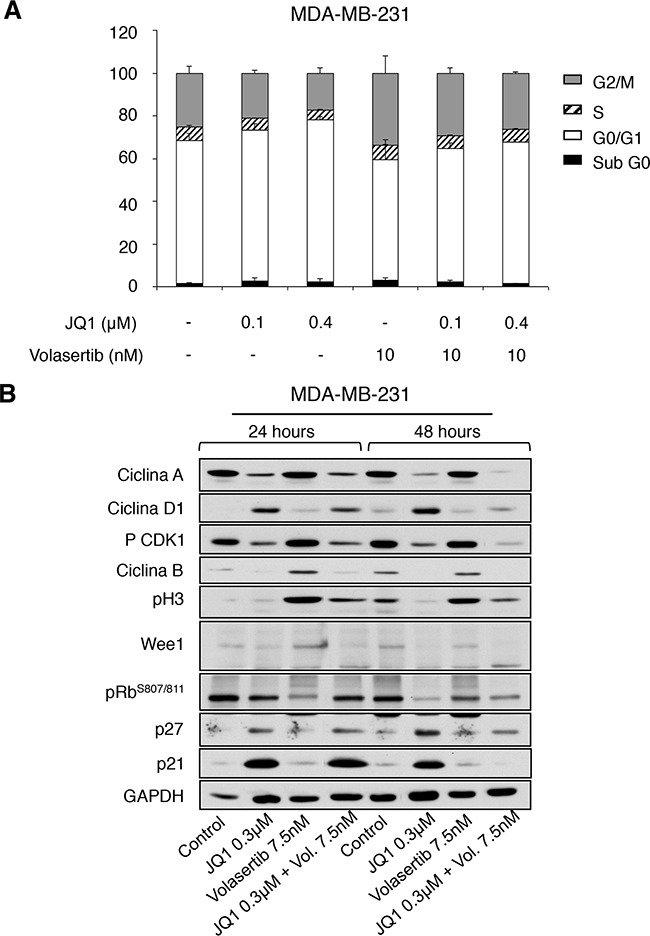
Effect of JQ1 and Volasertib on cell cycle in MDA-MB-231 **A**. Flow cytometry analysis of the effect of BET and polo-like kinase inhibitors alone or in combination on the cell cycle of MDA-MB-231. Cells were synchronized and treated with the indicated doses. Cell cycle progression was examined after 24 hours of treatment by flow cytometry using propidium iodide DNA staining. The histogram shows the percentage of cells in different phases of the cell cycle. **B**. Semi-quantitative analysis of cell cycle-related regulators. Protein levels of Cyclin A, Cyclin D1, pCDK1, Cyclin B, pH3, Wee1, pRB, p27 and p21 present in MDA-MB-231 cell line before and after incubation with JQ1, Volasertib, and a combination of drugs for 24 and 48 hours; were determined by Western-blotting.

To get insights into the effect of each agent on cell cycle, we synchronized MDA-MB231 with double thymidine, treated them with JQ1 and Volasertib, and stained the cells with propidium iodide at 24 hours. Treatment with JQ1 was able to induce a cell cycle arrest at G1 (Figure 4A). As expected, administration of a polo-like kinase inhibitor produced an arrest at G2/M. Combination of both compounds showed a mixed effect with a slightly increased in G1.

Biochemical evaluation of their effect showed an increase in p21, p27 and cyclin D1 expression levels at 24 and 48 hours by the BET inhibitor, indicative of G1 arrest; and an increase of the mitotic marker pH3 in cells treated with Volasertib (Figure 4B). The combination did not show relevant changes in cell cycle proteins beyond those observed with each agent alone, suggesting that their action is complementary, affecting components of different phases of the cell cycle.

**Figure 5:**
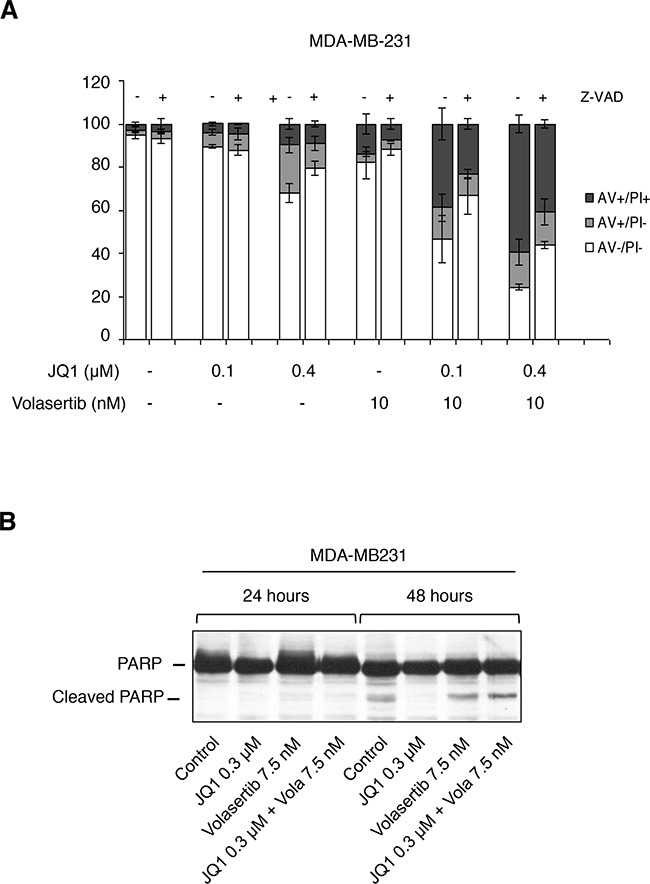
JQ1 and Volasertib induce cell death **A**. Pro-apoptotic activity of JQ1 alone and combined with Volasertib. MDA-MB-231 cells were treated with the indicated concentrations of drugs in the presence or absence of the pancaspase inhibitor Z-VAD-FMK for 48 h. Subsequently, cells were stained with Annexin V-DY-634 and analyzed by flow cytometry. The histogram represents the percentage of cells positive for Annexin V staining. **B**. Biochemical evaluation of PARP cleavage after drugs incubation. The protein levels of PARP on MDA-MB-231 treated with JQ1, Volasertib and the combination at different times (24 and 48 hours) were assessed by Western-blot analysis.

Next, we hypothesized that the combined inhibition of two phases of the cell cycle, would induce cell death more efficiently than the arrest of cells in a single phase. To do so, we stained cells with Annexin V after being treated for 24 and 48 hours. As can be seen in Figure 5A, an augmented effect with JQ1 and Volasertib was observed, compared with the individual treatments. The moderate inhibition of apoptosis with the pancaspase inhibitor Z-VAD-FMK, in addition to the slight presence of PARP degradation, suggested that the mechanism of induction of apoptosis with the drug combination includes caspase dependent and independent mechanisms (Figure 5A, B).

### Effect of the drug combination on the stem cell population

**Figure 6:**
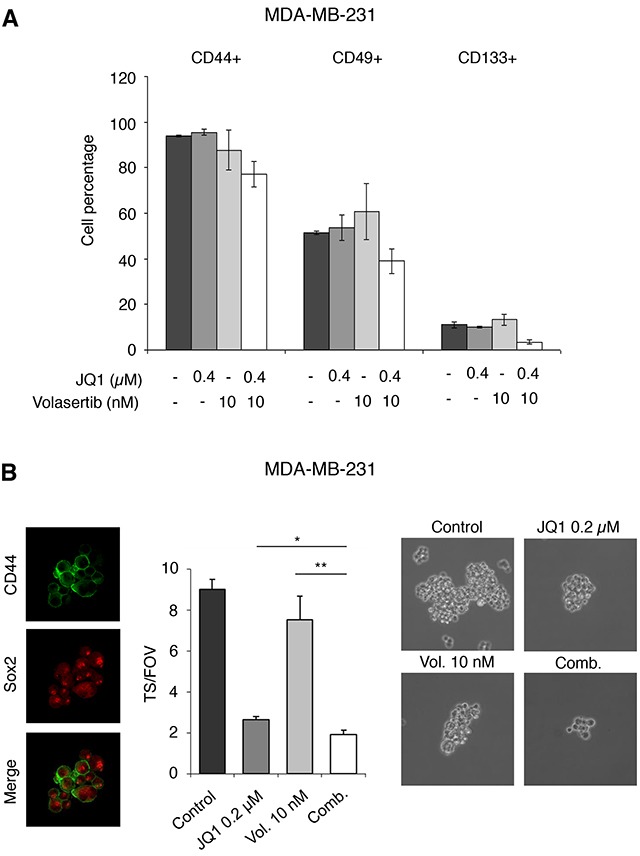
JQ1 and Volasertib reduce cell growth and expression of stem cell markers in tumorspheres **A**. MDA-MB-231 were treated with inhibitors at the indicated doses and 48 hours later CD44, CD49f, and CD133 surface expression on the whole cell population was examined by flow cytometry. The histogram represents the percentage of positive cells. **B**. CD44 and Sox-2 expression on MDA-MB-231-derived tumorspheres (TS) was evaluated by inmunofluorence using confocal microscopy, as described in material and methods. Secondary tumorsphere formation assays were performed to evaluate the effect of the compounds on the self-renewal capability of the cancer stem cell population.

As BET inhibitors have an effect on stem cell like populations [16, 20], we decided to explore the effect of BET inhibitors alone or combined with Volasertib on these properties. A reduction of several stem cell markers in MDA-MB231 grown as monolayers, including CD44, CD49 or CD133, was observed with the combination (Figure 6A). Next, we evaluated the effect of both agents of tumorsphere (TS) formation. To confirm the enrichment of cells with stem cell properties within the tumorspheres we used Sox2 and CD44 (Figure 6B). The combination of both agents was also able to reduce the formation of TS compared with each agent given alone (Figure 6B). These data clearly shows the pleiotropic effects of the combination that produces cell cycle arrest, induction of apoptosis, and has an effect on stem cell properties.

## DISCUSSION

In the present article, we describe a synergistic interaction of BET inhibitors in combination with polo-like kinase inhibitors in basal-like breast cancer. Cell division seems to be a deregulated function of this tumor type, and affecting it by targeting relevant kinases or by a combined effect by adding epigenetic modulators, seems to be an attractive approach.

We selected to inhibit the polo-like kinase because it was one of the upregulated kinases in our transcriptomic analyses, and its pharmacological inhibition had relevant effects on proliferation. The presence of “transcription factor” as a deregulated function in the functional analyses motivated us to explore the effect of BET inhibitors in association with the polo-like kinase inhibitor. The synergistic effect identified with the combination of both compounds in a panel of triple negative cell lines was unexpected. When getting insights into their effects, we observed through gene expression analyses that JQ1 also affected the cell cycle as a pathway, and specifically inhibited several kinases involved in the control of the mitotic process.

When assessing the effect of each agent alone or in combination, on different phases of the cell cycle, we observed that the BET inhibitor produced a cell cycle arrest at G1, while the polo-like kinase inhibitor induced cells to arrest at M. The individual effect of these agents on cell cycle has been previously described [11, 16]. Their combination showed a mixed effect on cell cycle but a clear induction of cell death. In summary, these experiments show that targeting cells by acting of different phases of the cell cycle is an attractive approach for the treatment of TNBC. The effect is cytotoxic rather than cytostatic, as observed with other targeted agents [6]. Therefore, this approach should be explored with other combinations.

On the other hand, as BET inhibitors act on the expression of several transcription factors, they could mediate their action by affecting several functions. In our analyses, they induced an arrest at G1 at the same time as reducing the levels of kinases involved in mitosis. Taken in consideration this pleotropic mechanism of action it is difficult to predict upfront in this disease which tumors will benefit from these agents. It would be expected that tumors with a higher proliferation rate would be more sensitive.

The effect of the drug combination on stem cell properties is not surprising. Inhibitors of mitosis produce a selection of populations more enriched in stem cell markers or in the so called “side population” [13]. Similarly, it has been described the effect of BET inhibitors through the inhibition of the JAK/STAT pathway on stem cells [16, 20] Therefore, these data further support their evaluation in combination.

Our study supports the use of epigenomic agents in combination with novel kinase inhibitors that act on the formation of the mitotic spindle. As new kinase inhibitors that act on mitosis are currently under development in TNBC [9–12], the identification of synergistic partners to improve efficacy is a main objective that can improve their clinical development.

Finally, we should acknowledge that not all TNBC are basal-like tumors. Indeed, our study used a dataset for basal-like tumors. In addition, TNBC is a heterogeneous disease, so it is not surprising that the synergistic effect was not observed in all cell lines evaluated. The identification of a biomarker that could predict response is a future task.

In conclusion, we describe for the first time the synergistic interaction between BET inhibitors and polo-like kinase inhibitors in basal-like breast cancers. Our results have important implications for the clinical development of these agents.

## MATERIALS AND METHODS

### Cell culture and drug compounds

MDA-MB-231, HS578T, BT549 were growth in DMEM and HCC3153 was growth in RPMI containing 10% fetal bovine serum (FBS). For non-adherent cultures of MDA-MB-231, tumorspheres were cultured with DMEM-F12 plus B27 supplement (2%, Thermo Fisher Scientific), bFGF (20ng/ml) and EGF (20ng/ml) (Sigma Aldrich). All media were supplemented with 100 U/mL penicillin, 100 μg/mL streptomycin and 2 mM L-glutamine and cells were maintained at 37°C in a 5% CO_2_ atmosphere. All cell lines were provided by Drs J Losada and A Balmain (originally from Dr. JW Gray's Laboratory, who in turn obtained them from the ATCC or from collection development in the laboratories of Drs S Ethier and A Gazdar to avoid errors occurring when obtained through ‘second-hand’ sources). In addition cells were analyzed by STR at the molecular biology unit at the Salamanca University Hospital.

The cell culture medium and supplements were obtained from Sigma Aldrich (St. Louis, MO). Cell lines were treated with different drugs: Bromodomain and extra-terminal proteins inhibitor (JQ1) and PLK1 inhibitor (Volasertib) were purchased from Selleckchem (Houston, TX).

### Whole genome transcription profiling and gene-set enrichment analyses

We used a public dataset (GEO DataSet accession number: GDS2250)[18.^19^] of mRNA level data from normal breast tissue and basal-like breast tumor to identify deregulated genes. This dataset does not include information about HER2 and Luminal subgroups. Affymetrix CEL files were downloaded and analyzed with Transcriptome Analysis Console (TAC) Software, developed by Affimetrix. For the evaluation of other breast cancer subtypes (HER2 enriched and Luminal) we used data contained at GSE45827.

Genes with different expression values from the control vs basal-like breast tumor were obtained. The list of genes was analyzed using gene set enrichment analyses DAVID Bioinformatics Resources 6.7 in order to identify functions of these genes. We used a adjusted p-value <0.05 to select the enriched gene-sets.

MDA-MB-231 cells were grown in DMEM with 10% of FBS and seeded to a final concentration of 500.000 cells/plate. On the following day, cells were treated with 500 nM JQ1 for 12h and 24h. Total RNA was extracted from purified cell populations using RNeasy Mini Kit (Qiagen, Valencia, CA, USA) according to the manufacturer's instructions. The RNA integrity was assessed using Agilent 2100 Bioanalyzer (Agilent, Palo Alto, CA). Labelling and hybridizations were performed according to protocols from Affymetrix. Briefly, 100 ng of total RNA were amplified and labeled using the WT Plus reagent kit (Affymetrix) and then hybridized to Human Gene 2.0 ST Array (Affymetrix). Washing and scanning were performed using *GeneChip* System of Affymetrix (GeneChip Hybridization Oven 645, GeneChip Fluidics Station 450 and GeneChip Scanner 7G).

Genes with different expression values from the control vs treated groups (12h, 24h) were obtained. The list of genes was analyzed using gene set enrichment analyses DAVID Bioinformatics Resources 6.7 in order to identify pathways modified by drug treatment. We used an adjusted p-value <0.05 to select the enriched gene-sets.

### Cell proliferation studies: MTT assays, drug combination analysis, colony-formation experiment and matrigel-embedded cultures

The effect of drugs on cell proliferation was assessed using MTT (3-(4, 5-dimethylthiazol-2-yl)-2, 5 diphenyltetrazolium bromide) screening assay, where MTT is reduced to purple formazan by the mitochondria of living cells. Increase in cell number is detected by augmented MTT metabolization, and decrease in cell number is reflected by decrease in MTT metabolization. Triple-negative breast cells were plated at a density of 10,000 cells per well in 48-multiwell plates and cultured overnight in DMEM or RPMI supplemented with 10% FBS and 5 mM glutamine. The next day, cells were treated for three days with increasing concentrations of JQ1 alone or in combination with Volasertib to plot the dose–response curves and for synergy studies, respectively. In parallel, cells were treated with increasing amounts of Volasertib, Alisertib, Docetaxel and AZ3146 at three-time points (24 h, 48 h and 72 h of incubation) to determine the IC50 value. After drug administration, the medium was replaced with 400 μL of fresh medium DMEM without phenol red containing MTT (0.5 μg/μL) and incubated for 45 minutes at 37°C. The medium was then removed and 200 μL of dimethylsulfoxide (DMSO) were added to each well. The plate was agitated in the dark for 5 minutes to dissolve the MTT-formazan crystals. The absorbance of the samples was recorded in a multiwell plate reader (BMG labtech).

To evaluate whether the combinations of JQ1 with Volasertib were synergistic, additive or antagonic, we used the Calcusyn Version 2.0 software (Biosoft, Ferguson, MO) and combinational index (CI) was determined. Thus, values < 1 represent synergistic effect on cell proliferation of the 2 drugs, values equal to 1 indicate additive effect of the drugs, and values > 1 represent an antagonistic effect.

To perform the colony-forming assay, cells were seeded at a density of 500,000 cells in 100 mm culture dishes, and the next day were treated with JQ1 (500 nM) or Volasertib (10 nM). For clonogenic combinations, MDA-MB-231 and HS578T were growth in presence of JQ1 (100 nM) plus Volasertib (10 nM). After 5 hours treatment, cells were trypsinized, resuspended in 5 ml of complete growth medium to perform serial dilutions 1/10 and seeded, in triplicate, in 6-multiwell plates for 10 days. Then, the medium was removed and the number of colonies was determined.

For three-dimensional cultures, MDA-MB-231 and HS578T cells were trypsinized and resuspended in growth medium containing 2% Matrigel. Then, cells were seeded at a density of 25.000 cells/ml in a 48-multiwell plate containing an underlying approximately 1 mm thick bed of Matrigel and incubated at 37°C. After overnight incubation, cells were exposed to JQ1 (200 nM) or Volasertib (7,5 nM) alone or in combination for 3 days. The assay included the daily visualization of cells under a light microscope to determine the total number of colonies per plate as well as to monitor the phenotype.

Unless otherwise indicated, all results are presented as the mean ± s.d. of triplicates of a representative experiment that was repeated at least three times.

### Flow cytometry

For cell cycle analysis, MDA-MB-231 cells were cultured in 60 mm culture dishes, grown to a density of 300.000 cells. After 24h, were synchronized with double thymidine block (2mM). Briefly, cells were exposed to thymidine for 18h, and then, after recovering in thymidine free medium for 9h, a second exposure was performed for another 18h. Then, cells were treated with JQ1 (100 and 400 nM), Volasertib (10 nM) or the combination of both drugs for 24h.

Cell monolayers were collected by pooling together the non-attached and attached cells and incubated in trypsin-EDTA, washed twice with cold PBS, fixed in ice cold 70% ethanol for 30 minutes and subsequently centrifuged at 5000 rpm for 5 minutes. Cell pellets were washed in PBS+2% BSA and treated with Propidium iodide/RNAse staining solution (Immunostep S.L., Salamanca, Spain) in the dark for 1 hour at 4°C.

For apoptosis and caspase assays, MDA-MB-231 cells were seeded at a density of 300.000 cells per 100-mm dish and 24 h later, pre-treated with 50 μM Z-VAD-ZMK pan-caspase inhibitor for 1 h before adding JQ1 (100 and 400 nM) and Volasertib (10nM), and its combinations for 72 hours. After drug exposure, adherent cells were trypsinized, pooled with the floating cells and washed twice with cold-PBS. Then, cells were stained with 5 μl of Annexin V-DT-634 (Immunostep S.L., Salamanca, Spain) and 3 μl of Propidium iodide (10 mg/ml) in 1x Binding Buffer (10 mM HEPES, pH 7.4, 140 mM NaOH, 2.5 mM CaCl_2_) for 1 hour at room temperature in the dark. Both early apoptotic (Annexin V-positive, PI-negative) and late (Annexin V-positive and PI-positive) apoptotic cells were included in cell death determinations.

For determination of stem cells markers, MDA-MB-231 cells were incubated for 1 h at 4°C with CD44 (10μL/sample), CD49f (2 μl/sample) and CD133 (10μL/sample) antibodies (Inmunostep, Salamanca, Spain) and then, surface expression was examined.

All samples were performed using a FACSCanto II flow cytometer (BD Biosciences) and the data analysis were performed using the FACS Diva software.

### Quantitative reverse-transcription PCR

mRNA of untreated and JQ1-treated (at 12 and 24 hours) cells were collected and the levels of the indicated genes were determined by quantitative reverse- transcription PCR. Briefly, total RNA was obtained from cells using RNeasy Mini Kit (Qiagen, Hilden, Germany) according to manufacturer's instruction. After extraction, concentration and purity were determined using a NanoDrop ND-1000 spectrophotometer (Thermo Fisher Scientific, USA) and, subsequently, 3 μg of total RNA was reversely transcribed using RevertAid H Minus First Strand cDNA syntesis Kit (Thermo Fisher Scientific, USA) in a thermocycler (Bio-Rad) under the following reaction conditions: 65°C for 5 min, 42°C for 60 min and 70°C for 10 min. The cDNAs were then subjected to a real-time PCR analysis using Fast SYBR Green Master Mix in StepOnePlus Real-Time PCR system (Applied Biosystems) according to the manufacturer's instructions. Primer sequences used were as follows: NEK2 forward 5′-GGAAGAGTGATGGCAAGATA-3′, and reverse 5′-CACTAGCCAGATCCCCTCCT-3, PBK forward 5′-AGTCCTGGGAGAGGGAGGAG-3′ and reverse 5′-CCCAGCGAGACCCTGCAGCT-3′, BUB1B forward 5′-AACCTTTAAGGCAAGGGCGG-3′, and reverse 5′-CCACCTTGAGGATAGTTCTG-3′, AURKA forward 5′-GCGGGTCTTGTGTCCTTCAA-3′, and reverse 5′-GCCAGTTCCTCCTCAGGATT-3′, and TTK forward 5′-GCCACCACAAGATGCAGAAA-3′, and reverse 5′-CAGGCACAACCAAATCTCGG-3′. An initial step was performed at 95°C for 10 min, followed by 40 cycles of 95°C for 15 sec and finished by 60°C for 1min. each sample was analyzed in triplicates and cycle threshold (Ct) values of transcripts was determined using StepOne Software v.2.1.

The Ct values were calculated using GAPDH as reference. Untreated MDA-MB231 and HS578T cells were used as control to calculate the Ct value and to determine the X-fold mRNA expression.

### Preparation of cell extracts and western-blotting

MDA-MB-231 cells were plated at a density of 700.000 cells/100 mm dish, maintained overnight in DMEM+10% FBS, and treated later with JQ1 (100 and 300 nM), Volasertib (7,5 nM) and the combination for 6, 12, 24 and 48 hours. After treatment, cells were washed with cold PBS and lysed in cold lysis buffer (20 mM Tris–HCl [pH 7.0], 140 mM NaCl, 50 mM EDTA, 10% glycerol, 1% Nonidet P-40, 1 μM pepstatin, 1 μg/mL aprotinin, 1 μg/mL leupeptin,1 mM phenylmethyl sulfonyl fluoride, 1 mM sodium orthovanadate). Then, insoluble material was removed by centrifugation. The protein concentration was determined using BCA (Bicinchoninic acid) protein assay kit (Sigma Aldrich).

For Western-blotting, 50 μg protein was resolved by 6%–15% sodium dodecyl sulfate polyacrylamide gel electrophoresis (SDS-PAGE) and transferred to polyvinylidene difluoride membranes (Millipore Corporation). Blots were blocked in 1x Tris-buffered saline (TBS, 100 mM Tris [pH 7.5], 150 mM NaCl, 0.05% Tween 20) containing 0.05% Tween 20 and 1% of bovine serum albumin for 1 hour and then incubated overnight with the following primary human monoclonal/polyclonal antibodies: anti-GAPDH, anti-Wee1, anti-cyclin B, anti-p21 (purchased from Santa Cruz Biotechnology, USA), anti-p(Y15)CDK1, anti-p27, anti-cyclin D1, anti-p(S807/811)RB (obtained from Cell Signalling Technologies, Beverly, MA, USA), anti-cyclin A and anti-p histone 3 (purchased from BD Biosciences, San Jose, CA, USA). Protein-bound primary antibodies were detected using horseradish peroxidase-coupled secondary antibodies (anti-rabbit or anti-mouse, obtained from Santa Cruz Biotechnology) diluted 1:5,000 in 1x TBS containing 0.05% Tween and incubated for 30 min at room temperature. Protein bands were detected using ECL Plus Western Blotting Detection System (GE Healthcare, Buckinghamshire, United Kingdom).

### Immunofluorescence

MDA-MB-231 cells grown on glass coverslips were fixed in 4% paraformaldehyde for 10 minutes. Cells were rinsed twice with PBS and blocked in PBS containing 0.1% Triton X-100 and 4% BSA for 1 hour, and subsequently incubated overnight at 4°C with anti-β-tubulin (1:250, Santa Cruz Biotechnologies) and anti-Nucleoporin p62 (1:200, BD transduction laboratories) primary antibodies. Cells were washed three times in PBS and incubated with an anti-mouse Alexa Fluor 568 (1:1000) antibody for 60 minutes. DAPI (300 nM) was added for 10 min and washed twice with PBS before mounting.

Fluorescence imaging of cells was performed using an epifluorescence inverted microscope (DMIRE-2, Leica) with a PlanApo 40x oil immersion objective. The excitation light source was a high-speed scanning polychromator with a Xe lamp (C7773, Hamamatsu Photonics). An emission filter wheel was controlled by a Lambda-10 device (Sutter Instruments). Images were acquired with an ORCA-FLASH 4.0 camera (C11440-22CU) and Aquacosmos 2.6 software (both from Hamamatsu Photonics) was used to control all devices.

For images of β-tubulin and Nucleoporin staining, samples were excited at 568 nm and emission was collected with emission filter 620/55 nm (center wavelength/bandwidth) through an excitation filter and triple-edge dichroic mirror (422/520/590 nm). The DAPI images were obtained exciting at 405 nm and using a 475/20 nm emission filter and a 455DRLP dichroic filter.

Alternatively, images were obtained in a Zeiss LSM 710 confocal microscope with a Plan Apo 63x oil immersion objective. Samples were excited at 405 nm and 543 nm and emission was collected between 498–535 nm and 587–666 nm to obtain DAPI and β-tubulin/ Nucleoporin p62 signals, respectively.

MDA-MB-231-derived tumorspheres (TS), were dropped on poly-lysine-treated slides for 1 min, before being fixed with 4% of PFA for 10 min at RT. TS were then permeabilized for 5 min with 0,1% Triton X-100 in PBS, washed and blocked with 3% BSA for 30 min. Then, slides were incubated for 1 hour with R-phycoerythrin (PE)-coupled CD44 (Inmunostep) and Sox-2 (Millipore). Sox-2 incubated slides were then washed with PBS and incubated with an anti-rabbit Alexa Fluor 568 antibody for 60 minutes. TS were again washed with PBS before mounting with Fluoroshield (Sigma). Fluorescence imaging of TS was performed using confocal microscopy (Zeiss Zeiss LSM 710) with a 63x oil immersion objective.

### Secondary tumorspheres formation assays

MDA-MB-231-derived tumorspheres (TS) were mechanically dissociated by up and down pipetting and 200.000 cells cultured in ultralow attachment 100 mm plates (Falcon) in the presence of JQ1 (200 nM), Volasertib (10 nM) or combination of both. The following day, TS were again dissociated. At 72 hours, newly formed TS were blindly counted on 25 random fields of view (FOV). TS/FOV was calculated from 3 independent experiments. Two-ways Student's test was used for the statistical analyses.

## SUPPLEMENTARY FIGURES AND TABLES


