# Supplementary material for: Targeting basal-like breast tumors with bromodomain and extraterminal domain (BET) and polo-like kinase inhibitors
Source: Oncotarget. 2017 Jan 3;8(12):19478–90. doi: 10.18632/oncotarget.14465 (PMC5386699; doi:10.18632/oncotarget.14465)
Supplement: Supplementary file 1 [file oncotarget-08-19478-s001.pdf]

Targeting basal-like breast tumors with bromodomain and extraterminal domain (BET) and polo-like kinase inhibitors

SUPPLEMENTARY TABLE

Supplementary Table 1: Expression of mitotic kinases in different breast cancer subtypes

GSE45827		NORMAL BREAST(n=11) vs BASAL SAMPLE (n=41)	NORMAL BREAST(n=11) vs HER 2 SAMPLE (n=30)	NORMAL BREAST(n=11) vs LUMINAL A (n=29)	NORMAL BREAST(n=11) vs LUMINAL B (n=30)	NORMAL BREAST(n=11) vs LUMINAL A+B (n=59)
**GENE SET**	**GENE NAME**					
204641_at	NEK2. NIMA (never in mitosis gene a)-related kinase 2	18.24	5.88	---	5.02	---
219148_at	PBK. PDZ binding kinase	12.07	9.8	---	6.61	4.32
204822_at	TTK protein kinase	11.91	11.33	---	6.06	---
208079_s_at	AURKA. Aurora kinase A	10.01	7.23	---	6.35	---
209464_at	AURKB. Aurora kinase B	4.06	4.63	---	---	---
209642_at	BUB1. Budding uninhibited by benzimidazoles 1 homolog	11.78	7.11	---	4.95	---
203755_at	BUB1B. Budding uninhibited by benzimidazoles 1 homolog beta	6.42	9.59	---	6.26	4.05
203213_at	CDC2. Cell division cycle 2, G1 to S and G2 to M	17.95	12.04	---	9.6	5.39
243831_at	MAPK6. Mitogen-activated protein kinase 6	6.27	---	--	---	---
202240_at	PLK1. Polo-like kinase 1	6.13	2.89	---	---	---
